# Cardiac Rehabilitation in Patients with Coronary Heart Disease—Challenges, Inequalities, and Opportunities for Global Health

**DOI:** 10.5334/gh.1480

**Published:** 2025-10-03

**Authors:** Vagner Madrini, Monica T. A. Albuquerque, Caio A. M. Tavares, Patricia O. Guimarães

**Affiliations:** 1 Hospital Israelita Albert Einstein, São Paulo, Brazil; 2 Instituto do Coração (InCor), Hospital das Clinicas HCFMUSP, Faculdade de Medicina, Universidade de São Paulo, São Paulo, Brazil

**Keywords:** cardiac rehabilitation, secondary prevention, health equity

## Abstract

Cardiac rehabilitation (CR) is a cornerstone of secondary prevention in coronary heart disease, supported by robust evidence and classified as a Class I recommendation in international guidelines. Despite its proven benefits in reducing morbidity, mortality, and improving quality of life, CR remains strikingly underutilized worldwide, revealing a paradox of high-level evidence with low-level implementation. The INTERASPIRE study highlights this global gap, showing that many patients are neither referred nor adhere to CR programs, and that profound inequities persist across regions and socioeconomic groups. These findings underscore systemic failures in translating guideline recommendations into practice, driven not only by structural limitations but also by physician referral patterns, patient awareness, and health system priorities. Addressing this gap requires investment in infrastructure, equitable referral strategies, standardization of program content, and innovative delivery models such as telehealth. Ensuring universal access to CR is both a clinical imperative and a matter of health equity, with the potential to transform outcomes for patients with cardiovascular disease worldwide.

Cardiac rehabilitation (CR) is widely recommended by international guidelines for patients with cardiovascular disease (1,2,3), with robust evidence of its effectiveness in reducing morbidity and mortality associated with coronary heart disease (CHD) (4). Importantly, CR is not merely confined to immediate post-acute coronary care, but plays a fundamental role across the different phases of CHD—ranging from inpatient service, supervised ambulatory programs, and long term maintenance (5). CR represents one of the few interventions in cardiovascular medicine that may deliver multidimensional benefits: risk factor modification, improved adherence to medications, enhanced quality of life, and reduction in hospitalizations (6).

Yet, despite this overwhelming body of evidence and its Class I recommendation in guidelines, real-world data reveals a striking paradox: CR programs remain underutilized globally (7). The INTERASPIRE study gathered data from 88 hospitals in 14 countries across six World Health Organization (WHO) regions, and demonstrated inadequate implementation of guideline-recommended interventions for secondary prevention—particularly CR. It was seen that 91% of patients did not attend more than half of the advised sessions (8). The study by McEvoy et al. dug deeper into the INTERASPIRE data and provided a comprehensive assessment of this paradox. Its findings exposed the challenges and inequalities of CR implementation worldwide. Although ideally CR should be available for all patients with CHD, only one-third of the 4548 participants were advised to participate in a CR program, and around 20% attended at least half of the sessions. Even in centers where CR was available—76 out of 88 participating hospitals—referral rates remained unacceptably low (38,5%), underscoring the gap between evidence-based recommendations and routine care.

The disparity between high- and low-income countries further compounds this issue: referral rates ranged from just 4% in Kenya to nearly 70% in Poland, reflecting alarming inequities in access to cardiovascular care. In Colombia and Indonesia, more than 50% of patients were referred to CR programs, while in Portugal and Singapore, these rates were below 50%, demonstrating that a country’s economic status is not the only barrier. Indeed, cost-effectiveness has already been demonstrated, and are driven mainly by reduction in new events, hospitalizations and additional interventions (9).

The presented analysis also captured important information regarding differences in referral to CR according to patient profiles. Individuals aged 65 years or older, those with a lower educational level, and unemployed status were less advised to participate in CR programs. Interestingly, older patients, when referred, had higher attendance rates, showing that this vulnerable population could benefit from higher referrals. The efficacy of CR in the elderly has also been proved to be similar to that in the young (10). Altogether, these findings highlight a crucial opportunity for improvement. Given the breadth of CR’s impact across cardiovascular diseases, its underuse represents a failure of health systems to deliver an intervention with substantial and well-documented benefits.

Access to CR is heterogeneous worldwide, as availability varies considerably across regions and largely reflects systemic differences in health infrastructure and policy. In general, high-income countries demonstrated greater referral rates, while in many low- and middle-income countries, structural and financial limitations hindered access. Several factors are associated with referral and adherence to CR—Age, education, employment status, and income influenced adherence—confirming that socioeconomic barriers remain decisive obstacles to participation. Unlike previous studies, this analysis showed no difference in referral rates between men and women (11).

CR also provided a significant impact on health measures. Attendance was associated with reductions in smoking and physical inactivity, improved blood pressure and low-density lipoprotein (LDL) cholesterol control, and higher adherence to cardioprotective therapies. However, persistent smoking, obesity, and physical inactivity remained highly prevalent, underscoring the need for more comprehensive, multifaceted CR programs.

There is also marked variability in the content of CR programs. Striking heterogeneity in program components—ranging from exercise to psychological support—limits comparability and calls for global efforts toward program standardization.

The paradox highlighted by INTERASPIRE—high-level recommendations with low-level implementation—demands urgent attention. Unlike other interventions in cardiology that may hold weaker evidence yet see broader adoption, CR remains critically underutilized despite its proven effectiveness across the entire cardiovascular spectrum. It is important to note that CR benefits extend to patients with heart failure (HF) (17), arrhythmias (18), valvular disease and beyond. The missed opportunity is particularly glaring in health systems where CR services exist but referral remains infrequent, suggesting that barriers lie not only in availability but also in physician practices, patient awareness, and systemic prioritization. Automatic referral along with a strong recommendation by the physician in charge may represent a turning point in this matter (12).

The low rates of attendance to CR programs are also alarming. Among those referred, 42.8% attended < 50% of the proposed sessions or did not participate at all. Strategies and policies are needed to understand and address the causes of absence such as those related to insurance coverage and coparticipation. Importantly, patient awareness of the clinical benefits of CR should be prioritized. To address this huge gap, national cardiology societies, patient associations, and health authorities must advocate for substantial investment in CR infrastructure and standardized program development, particularly in resource-limited settings. Involving insurance companies in the discussion is also imperative. Telehealth, mobile device-based or home-based interventions (13,14,15), are also effective and may be considered depending on patient preference and availability, especially—but not only—in terms of the long-term maintenance (16) and to surpass geographic inaccessibility.

The INTERASPIRE study exposed the scenario of underutilization of CR, despite its clinical benefits in cardiovascular disease. This paradox represents a failure of implementation of guideline-recommended therapies in clinical practice. Expanding CR through standardized programs, reducing socioeconomic barriers, and strengthening referral pathways are imperative steps to close this gap. Ultimately, ensuring better access to CR is an issue of health equity, as it holds the potential to improve outcomes for patients with CHD worldwide.
